# Implementation of an interprofessional collaboration in practice program: a feasibility study using social network analysis

**DOI:** 10.1186/s40814-020-00746-3

**Published:** 2021-01-06

**Authors:** Linda C. Smit, Jeroen Dikken, Nienke M. Moolenaar, Marieke J. Schuurmans, Niek J. de Wit, Nienke Bleijenberg

**Affiliations:** 1 Research Centre for Healthy and Sustainable Living, University of Applied Sciences Utrecht, 3584 CS Utrecht, The Netherlands; 2 Faculty of Health, Nutrition and Sport, The Hague University of Applied Sciences, The Hague, The Netherlands; 3 Dutch Inspectorate of Education, Utrecht, The Netherlands; 4 Education Center, UMC Utrecht Academy, University Medical Center Utrecht, 3508 GA Utrecht, The Netherlands; 5 Department of General Practice, Division Julius Center for Health Sciences and Primary Care, University Medical Center Utrecht, Utrecht, The Netherlands; 6 Department of Nursing Science, Julius Center for Health Sciences and Primary Care, University Medical Center Utrecht, Utrecht, The Netherlands

**Keywords:** Interprofessional education, Interprofessional collaboration, Social Network Analysis, Primary care

## Abstract

**Background:**

Due to multimorbidity and geriatric problems, older people often require both psychosocial and medical care. Collaboration between medical and social professionals is a prerequisite to deliver high-quality care for community-living older people. Effective, safe, and person-centered care relies on skilled interprofessional collaboration and practice. Little is known about interprofessional education to increase interprofessional collaboration in practice (IPCP) in the context of community care for older people. This study examines the feasibility of the implementation of an IPCP program in three community districts and determines its potential to increase interprofessional collaboration between primary healthcare professionals caring for older people.

**Method:**

A feasibility study was conducted to determine the acceptability and feasibility of data collection and analysis regarding interprofessional collaboration in network development. A questionnaire was used to measure the learning experience and the acquisition of knowledge and skills regarding the program. Network development was assessed by distributing a social network survey among professionals attending the program as well as professionals not attending the program at baseline and 5.5 months after. Network development was determined by calculating the number, reciprocity, value, and diversity of contacts between professionals using social network analysis.

**Results:**

The IPCP program was found to be instructive and the knowledge and skills gained were applicable in practice. Social network analysis was feasible to conduct and revealed a spill-over effect regarding network development. Program participants, as well as non-program participants, had larger, more reciprocal, and more diverse interprofessional networks than they did before the program.

**Conclusions:**

This study showed the feasibility of implementing an IPCP program in terms of acceptability, feasibility of data collection, and social network analysis to measure network development, and indicated potential to increase interprofessional collaboration between primary healthcare professionals. Both program participants and non-program participants developed a larger, more collaborative, and diverse interprofessional network.

**Supplementary Information:**

The online version contains supplementary material available at 10.1186/s40814-020-00746-3.

## Introduction

With rapid population aging, the provision of care to older people with complex health issues resulting from multimorbidity and geriatric problems is a major challenge [1]. In the Netherlands, two-thirds of people aged 65 years and older are experiencing multimorbidity and geriatric problems. It is estimated that by 2050, 33.2% of the population will be aged 60 years and older [2]. Currently, 94% of older people in The Netherlands live at home, and their complex conditions need medical- and social-care solutions. For these solutions, interprofessional collaboration in practice (IPCP) between healthcare professionals is essential [3, 4].

Interprofessional collaboration has been defined as follows: an evolving interpersonal process, involving a diverse team of healthcare and other community providers who interdependently engage in frequent communication and shared decision-making, for the purposes of providing optimal health and social care services to community-living older adults and their families [5]. IPCP in healthcare occurs when multiple health workers from different professional backgrounds provide comprehensive services by working with patients, their families, caregivers, and communities to deliver the highest quality of care across settings [1]. Community care, however, is often provided by a heterogeneous workforce consisting of professionals by different levels of education working in different organizational structures that may hamper the ability to collaborate effectively [6, 7]. Interprofessional education (IPE) can support healthcare teams by utilizing the individual skills of their members, sharing case management, providing better health services to patients and the community, and improving patient outcomes [1, 8, 9]. IPE occurs when two or more professionals learn with, from, and about each other to improve collaboration and quality care [10]. Most IPE focuses on academic settings, and acute, and long-term care sectors [11]. Little is known about IPE to enhance interprofessional collaboration in practice for community-living older people [6, 12]. A pilot IPE program for general practitioners (GPs) and practice nurses from different community districts evaluated the effect of the IPE program for these professionals and reported that an IPE for professionals with different educational backgrounds (GPs and practice nurses) is feasible and adds value to the redefining of tasks and responsibilities among GPs and practice nurses [13]. However, studies examine the implementation of an IPCP program for primary care healthcare professionals from the medical and social domains are lacking. Therefore, a feasibility study was initiated to examine the implementation of an IPCP program [14] for healthcare professionals from the medical and social domains to enhance interprofessional collaboration. The aim of this study is to examine the feasibility of the implementation of an IPCP program in three community districts to determine its potential to increase interprofessional collaboration between primary healthcare professionals caring for older people. The feasibility objectives were as follows: (1) to determine the acceptability of the IPCP program, (2) to determine whether data can be collected during the implementation of an IPCP program to construct networks in a meaningful way, and (3) to examine the possibility of measuring network development in terms of the number, reciprocity, value, and diversity of contacts between healthcare professionals in three community districts.

## Method

### Study design

**Fig. 1:**
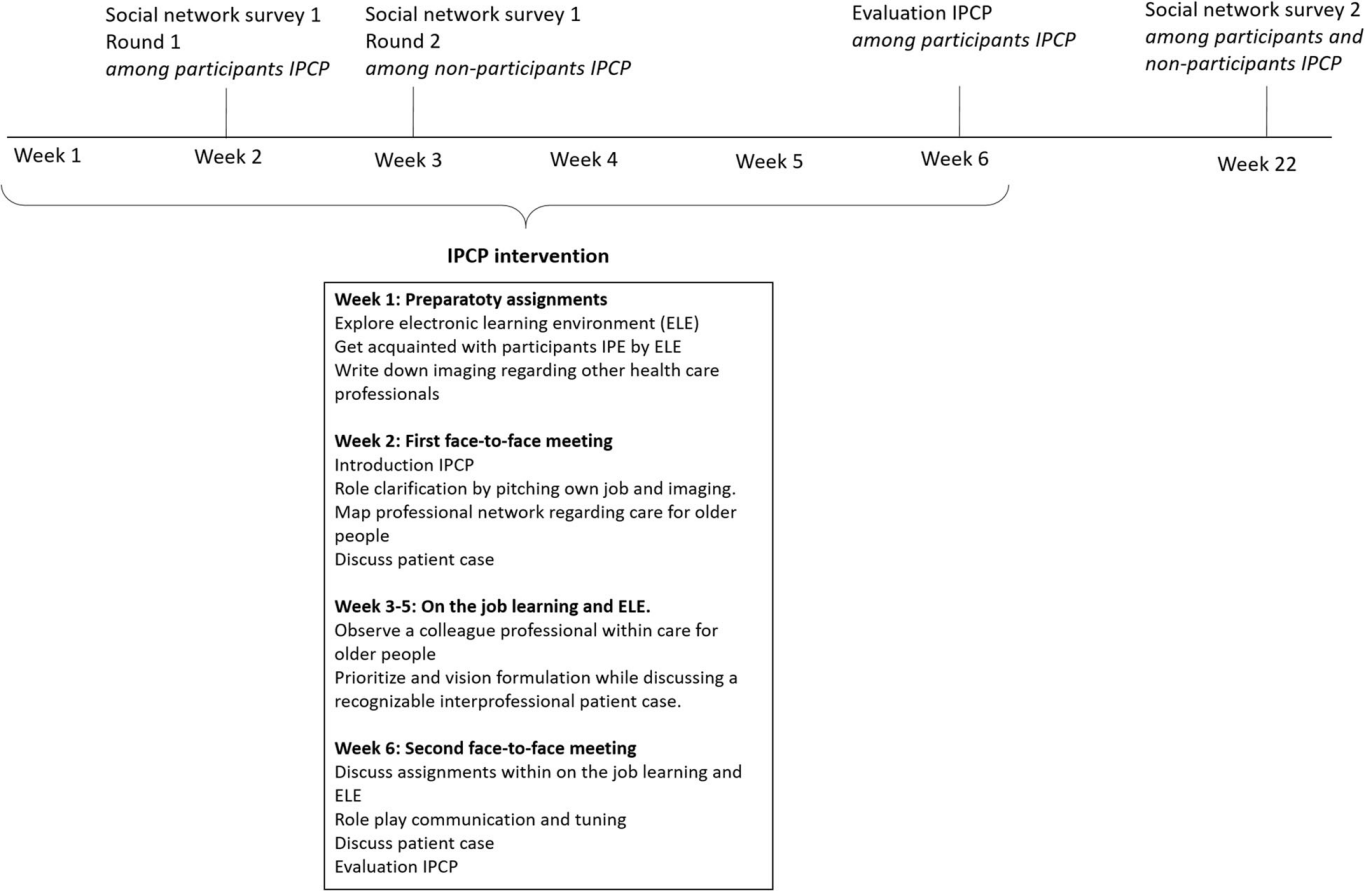
Study design and overview of IPCP program

We performed a pre-post study to examine the feasibility of implementing a previously developed IPCP program in three community districts. Figure 1 provides an overview of the study design and elements of the IPCP program.

### Participants and setting

Participants who were attending the IPCP program (“programme participants”) were primary healthcare professionals delivering care to community-living older people in three community districts in the city of Utrecht (350,000 inhabitants), the Netherlands. Participants included GPs, practice nurses, district nurses, social workers, physiotherapists, and pharmacists. In addition, “non-programme participants” were included. Non-program participants were professionals who did not participate in the IPCP program but only participated with consent in the social network data collection.

### IPCP program

The IPCP program was developed to enhance interprofessional collaboration among primary healthcare providers and was co-created by professionals from clinical practice, education, and research. The developmental process of the IPCP program has been described elsewhere [14]. A development team discussed the competencies of interprofessional collaboration [15], resulting in three main themes as the basis for the IPCP program, namely, role identity, shared vision, and communication. Based on these themes, learning objectives and activities were developed. The IPCP program included sixteen study hours and covered 6 weeks, and consisted of face-to-face meetings, online learning, and on-the-job learning (see Fig. 1). This blended learning approach was chosen to fit the diverse target group and for its positive effect on knowledge acquisition among health professionals [16]. A partner in developing the IPCP program, (as described elsewhere) [14], which is also an organization that offers educational training and guidance on collaboration in healthcare practices in the community, acted as independent coordinator in recruiting participants. Information leaflets were provided by the organization to the medical and social healthcare professionals as well as the regional coordinators. Professionals in the IPCP program participated voluntarily and free of costs because of the nature of the IPCP program as a feasibility study.

### Feasibility outcomes

The acceptability of the IPCP program was defined as (1) the views on the learning experience and its interprofessional nature, and (2) the acquisition of knowledge and skills linked to interprofessional collaboration indicated by the program participants [17].

The feasibility of data collection and analysis was determined by collecting and measuring network development regarding the interprofessional collaboration between professionals working in the same district. To compare the community’s collaboration networks, we assessed network development by the number of contacts with other professionals, the extent to which contacts are reciprocal, the diversity of contacts, and the perceived value of contacts.

### Data collection and measurement

The acceptability of the IPCP program was, after delivery, evaluated among 22 program participants using a self-reported questionnaire. The questionnaire, originally developed by the Expertise Center for Education and Training located at the Utrecht Medical Center (The Netherlands), was adapted to the context of IPCP and included two concepts based on the adapted framework of Kirkpatrick for interprofessional education [17]. First, participants’ satisfaction with the program was assessed by asking about perceptions of the content, organization, teaching, materials, and online environment of the IPCP program. Second, the applicability of the IPCP content was evaluated by asking about perceptions in acting toward fellow professionals and in applying knowledge and skills gained from the program. In total, the questionnaire involved 20 questions in which several measurement scales were used including 1-10 scales (4 questions, with a higher score indicating a higher appreciation), yes–a little–no scales (11 questions), and insufficient, sufficient, more-than-sufficient, good, very-good scales (5 questions).

Interprofessional collaboration was measured among program participants (*N* = 22) and non-program participants (*N* = 33; *N*_total_ = 55) using a social network survey. The IPCP program was delivered to a maximum of ten professionals in each district. The nature of the IPCP program was to enhance interprofessional collaboration in which we expect a spill-over effect of the IPCP program. To capture this spill-over effect, non-program participants were also included. A social network survey was administered at two-time points (see Fig. 1). At both time points, we posed the following network question: “out of all the primary healthcare professionals in the community, with whom do you collaborate regarding care for community-living older people?” [18]. At time point 1, data were collected in two rounds.

#### Round 1—Collecting data from IPCP program participants (ring 1)

**Fig. 2:**
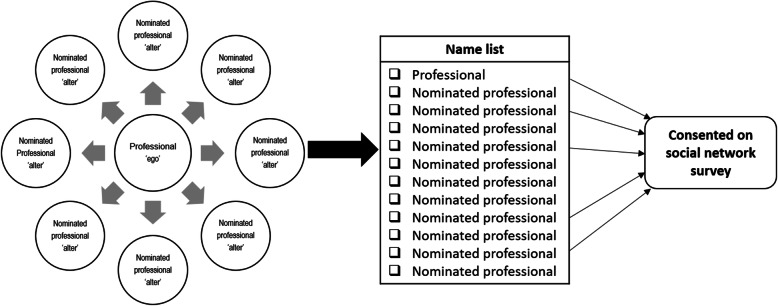
Diagrammatic representation of the network theory and data collection. Nominated professionals received an email with the social network survey; returning the survey was considered to indicate consent to participate in the study

The professionals who participated in the IPCP program (called “the first ring” of network members) were asked to provide a list of all primary healthcare professionals with whom they collaborate with about care for community-living older people [18]. In addition, we asked the program participants to indicate the value of these contacts on a scale of 1-10. Using these data, we visualized ego-networks for each participant (the so-called “ego”) and his/her contacts (the so-called “alters”). These contacts formed “the second ring” of network members surrounding each ego [19, 20]. Figure 2 provides a diagrammatic representation of the network theory and data collection.

#### Round 2—Collecting data from the program participants’ contacts in the district (ring 2)

Using the data collected in the first round, a list of names including all program participants (egos) and their nominations (alters) was created, resulting in a single, comprehensive list of potential healthcare professionals who may collaborate with each other in each community district. Subsequently, all professionals from this list were invited to complete, and thereby gave their consent for, the survey (see Fig. 2). We asked the participants to indicate with whom they collaborated in regard to care for community-living older people and how they value each of their contacts on a scale (1-10).

At the second time point, we again used this comprehensive list of names to delineate the community’s collaboration networks.

In each community district, between 27% and 40% of all nominated healthcare professionals responded and consented to the social network survey during round 2 of the data collection. The response rate also indicates the unit non-response, which is defined as completely missing for healthcare professionals for whom all outgoing contacts of the professional are missing but not the incoming contacts. This is because professionals who did respond also nominated the non-responding professionals as professionals with whom they work [21]. The collected data, therefore, were divided into three data categories: (1) program participants, (2) non-program participants, and (3) non-consented professionals, further described as rings 1, 2, and 3.

### Network development

#### Number of contacts

The number of contacts was calculated for each professional as the number of professionals with whom the participant indicated as collaborating with regarding care for older people (out-degree) [19]. A social network diagram was used to visualize the number of contacts between professionals. The professionals were visualized as colored squares of which the color indicates the ring 1, 2, or 3, in which the professionals were categorized. To compare the networks across community districts, we also calculated the network measures average degree of contacts, density, and E-I index [19]. The average degree of contacts was calculated as the average out-degree for each community district. The network’s density was calculated as the proportion of existing relationships out of the maximum number of relationships possible in the network. The denser the network, the more professionals collaborate with one another. The value of density varied between 0 (no relations in the network) and 1 (all actors are connected to each other). Finally, the E-I index was calculated for each network to determine whether ring 1 and ring 2 differed in their choice of alters. For instance, did ring 1 mainly increase collaboration with other ring 1 participants, or did they also increase collaboration with ring 2 members? And did ring 2 members also increase interprofessional collaboration, even though they did not participate directly in the IPCP program? The E-I index ranges from −1 (all contacts are internal to the group) to +1 (all contacts are external to the group).

#### Reciprocity of contacts

Reciprocity of contacts was calculated as the ratio of the number of pairs who shared a reciprocal network connection, (i.e., they both chose the other to collaborate with) relative to the number of pairs within any given contact. A high level of reciprocity reflects a high level of reciprocal collaboration between professionals in a district [19]. Reciprocal contacts of the professionals were visualized in a social network diagram displaying reciprocal and one-sided contacts between professionals for each district.

#### Diversity of contacts

Diversity of contacts was calculated as the extent to which contacts in the community district transcend the different backgrounds in disciplines (see Table 1) [18, 19]. The score for diversity in relationships can vary between 0 and 1. A high score indicates that the healthcare professional collaborates with healthcare professionals from a wide variety of disciplines (heterogeneity) while a low score indicates that the healthcare professional mainly chooses to collaborate with others from the same discipline (homogeneity).

**Table 1:** Definition and formula for diversity of contacts

Heterogeneity indicates, per healthcare professional; *i*, the degree of the number of relationships outside their own discipline in relation to the number of all possible different disciplines with which they are in contact. The number of times healthcare professional; *i*, has been chosen by the other healthcare professionals is considered (in-degree), because this parameter has a higher reliability with reality than the relationships indicated by the healthcare professional themselves (out-degree). Diversity is thus determined on the basis of two components: *P*_iR_, the proportion of healthcare professions *i*’s relationships with members of other disciplines, *R*_divi_, regarding all relationships of healthcare professions *i R*_i_; and *D*_iD_, the number of diverse disciplines with which healthcare professions; *i*, has contact outside of his own discipline, *D*_divi_, regarding all disciplines within the network minus the own discipline of healthcare professions, *i D*_i_: *P*_iR_ = (*R*_i_ − *R*_divi_)/*R*_i_ *D*_iD_ = *D*_divi_/*D*_i_ For every healthcare professional within the network, diversity is defined as: *H*_i_ = ΣP_iR_ × *D*_iD_ When a healthcare professional has relationships within all professionals, and as many professionals speak outside their own professionals as within their own professional, then *P*_iR_ = 0.5 and *D*_iD_ = 1. The diversity of relationships for this healthcare professional, *H*_i_, is 0.5/1 = 0.5. The score for diversity in relationships can vary between 0 and 1.	

#### Value of contacts

For each professional, we calculated the average of the value that s/he placed on each network contact. The score for the value of contact can vary between 1 and 10. A high score indicated that healthcare professionals highly appreciated their collaboration with that specific professional while a low score indicated a low appreciation for the collaboration.

### Sample size

During the development process of the IPCP program, it was decided to include seven to ten program participants per community district to achieve a high degree of interaction between professionals during the implementation of the program. The interaction between professionals was important to address the three main themes of the program, role identity, shared vision, and communication. A convenience sample of 22 program participants participated and consented to participate in this study. Non-program participants were included as well in determining the network development of each community district. Due to scattered healthcare organizations in the districts, it was very difficult to generate a name roster of all professionals per community district. By combining the snowball method (using the program participants) and a fixed-list selection of names, we obtained access to each whole community of healthcare professionals of which 33 non-program participants consented to participate in our study [19].

### Social network analysis

All social network measures were calculated and analyzed using UCINET 6.6, a network analysis program used for descriptive and inferential network statistics [20]. To determine a significant increase in the value and diversity of contacts, a paired *T* test was performed using the SPSS software version 24 for Windows (IBM SPSS Statistics, IBM Corporation, Armonk, NY).

## Results

Twenty-two participants participated in the IPCP program, and a total of 55 program and non-program participants were included in the data analysis (see Table 2).

**Table 2:** Healthcare professionals included in the study (*N* = 55)

	Total		District 1		District 2		District 3	
**Program participants**	***N*** **= 22**		***N*** **= 7**		***N*** **= 8**		***N*** **= 7**	
General practitioner, *n* (%)	3	13.6	1	14.3	1	12.5	1	14.3
Practice nurse, *n* (%)	1	4.6	1	14.3	0	0.0	0	0.0
Physiotherapist, *n* (%)	1	4.6	0	0	1	12.5	0	0.0
Social care worker, *n* (%)	5	22.7	1	14.3	2	25.0	2	28.6
Social care prescriber, *n* (%)	4	18.2	2	28.6	1	12.5	1	14.3
District nurse, *n* (%)	6	27.3	2	28.6	2	25.0	2	28.6
Pharmacist, *n* (%)	2	9.1	0	0	1	12.5	1	14.3
**Non-program participants**	*N* = 33		*N* = 9		*N* = 11		*N* = 13	
General practitioner, *n* (%)	5	15.2	1	11.1	1	9.1	3	23.1
Practice nurse, *n* (%)	5	15.2	2	22.2	1	9.1	2	15.4
Physiotherapist, *n* (%)	1	3.0	0	0.0	0	0.0	1	7.7
Social care worker, *n* (%)	10	30.3	2	22.2	5	45.5	3	23.1
Social care prescriber, *n* (%)	3	9.1	1	11.1	1	9.1	1	7.7
District nurse, *n* (%)	4	12.1	1	11.1	1	9.1	2	15.4
Pharmacist, *n* (%)	2	6.1	0	0.0	1	9.1	1	7.7
Specialist geriatric medicine, *n* (%)	1	3.0	1	11.1	0	0.0	0	0.0
Dietician, *n* (%)	1	3.0	1	11.1	0	0.0	0	0.0

### Acceptability of the IPCP program

The content of the IPCP program was experienced as instructive in 81% of the program participants and contributed to an enhanced interprofessional collaboration with an average score of 7.7 out of 10 (sd 1.0). Approximately 86% of the program participants indicated to act differently toward fellow professionals after attending the program, and 95% of the program participants indicated that they were able to apply the knowledge and skills of the program in practice. The participants valued the IPCP program with an average of 7.6 out of 10 (sd 1.0). For detailed information regarding the results of the questionnaire see Additional file 1.

### The number of contacts between professionals

**Fig. 3:**
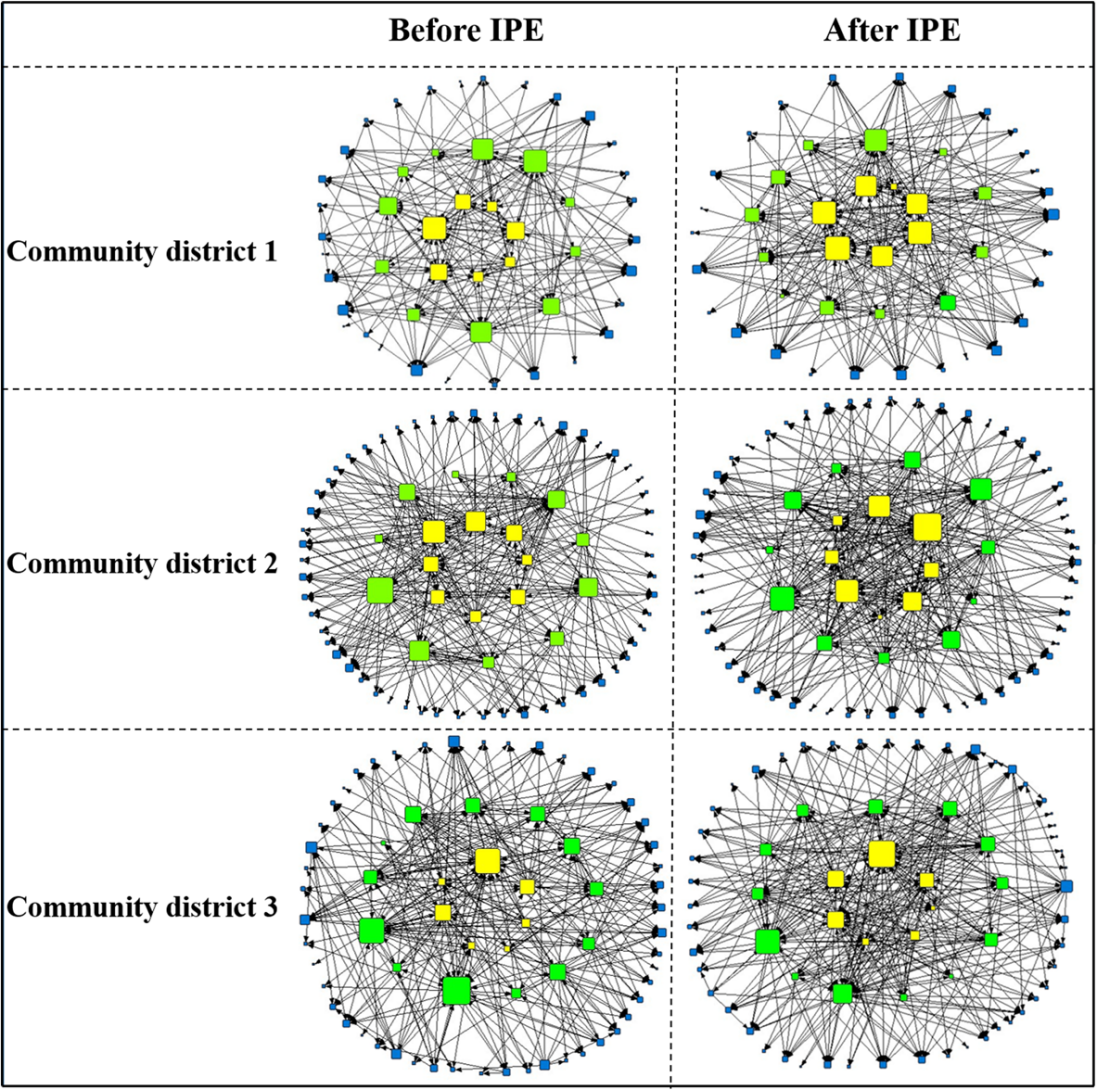
Community district collaboration networks before and after the IPCP program. Yellow squares, healthcare professionals in ring 1; green squares, healthcare professionals in ring 2; blue squares, healthcare professionals in ring 3. The larger the square the higher the number of professionals with whom the participant indicated as collaborating with regard care for older people (based on the out-degree of contacts). The black lines reflect a contact between professionals

The community’s collaboration networks before and after the IPCP program suggested that collaboration networks developed in each community district (see Table 3). In all districts, an increase in the number of contacts among the program participants was observed (ring 1). In district 1, ring 1 reported on average 4.8 contacts before and 7.5 contacts after the IPCP program. In addition, the results suggest increased collaboration between IPCP participants and other professionals in the district that did not participate in the IPCP program (rings 2 and 3). Figure 3 visualizes the increase in contacts over time for rings 1, 2, and 3.

**Table 3:** Descriptive statistics

	District 1		District 1		District 2		District 2		District 3		District 3	
	Rings 1 and 2		Rings 1, 2, and 3		Rings 1 and 2		Rings 1, 2, and 3		Rings 1 and 2		Rings 1, 2, and 3	
	*N* = 16		*N* = 49		*N* = 19		*N* = 75		*N* = 20		*N* = 69	
	T0	T1	T0	T1	T0	T1	T0	T1	T0	T1	T0	T1
**Number of contacts**												
Avg. degree	5.13	7.56	5.79	7.00	4.00	5.53	4.25	4.31	4.81	6.20	4.68	5.26
Density (%)	34	51	12	15	22	31	6	6	19	33	7	8
E-I index												
Ring 1												
Internal avg. degree	4.8	7.5			3.6	5.1			2.0	3.8		
Internal contacts	26	38			42	56			8	14		
External contacts	32	44			28	43			38	51		
Ring 2			**N.A.**				**N.A.**				**N.A.**	
External avg. degree	4.0	5.5			2.4	3.7			2.3	3.2		
Internal contacts	20	22			24	24			102	98		
External contacts	32	44			28	43			38	51		
E-I index												
E-I index ring 1	0.10	0.07			−0.20	−0.13			0.65	0.57		
E-I index ring 2	0.23	0.33			0.08	0.28			−0.46	−0.32		
**Collaboration**												
Reciprocity (%)	49	64	**N.A.**		25	27	**N.A.**		40	53	**N.A.**	
**Diversity of contacts**												
Mean	0.32	0.42			0.36	0.42			0.26	0.32		
(SD)	(0.17)	(0.14)	**N.A.**		(0.15)	(0.19)	**N.A.**		(0.17)	(0.14)	**N.A.**	
Paired *T* test												
t *p value*	−4.39	***< 0.001***			2.05	*0.055*			0.91	*0.371*		
**Value of contacts**												
Mean			7.3	7.7			7.6	7.8			7.5	7.5
(SD)	**N.A.**		(0.67)	(0.47)	**N.A.**		(0.61)	(0.65)	**N.A.**		(0.64)	(0.58)
Paired *T* test												
t *p value*			−1.28	*0.209*			−2.35	***0.022***			−1.12	*0.267*

In district 1, program participants reported an increase in contacts to non-program participants (from an average of 4.0 contacts before to 5.5 contacts after the IPCP program). An examination of the change in network density before and after the IPCP program suggests that in all three districts, the network density increased after the IPCP program. For example, for district 1, density increased from 34 to 51%. Before the program, roughly a third of all potential connections among the healthcare professionals were actually present within a district, which increased to about half of all connections after the program. In other words, after the program, the professionals in the district tended to collaborate with more and other professionals across the district. This increase in collaboration not only held for participants in the IPCP program but also extended to non-program participants, as expressed by an increased E-I index for all districts.

### Reciprocity of contacts

**Fig. 4:**
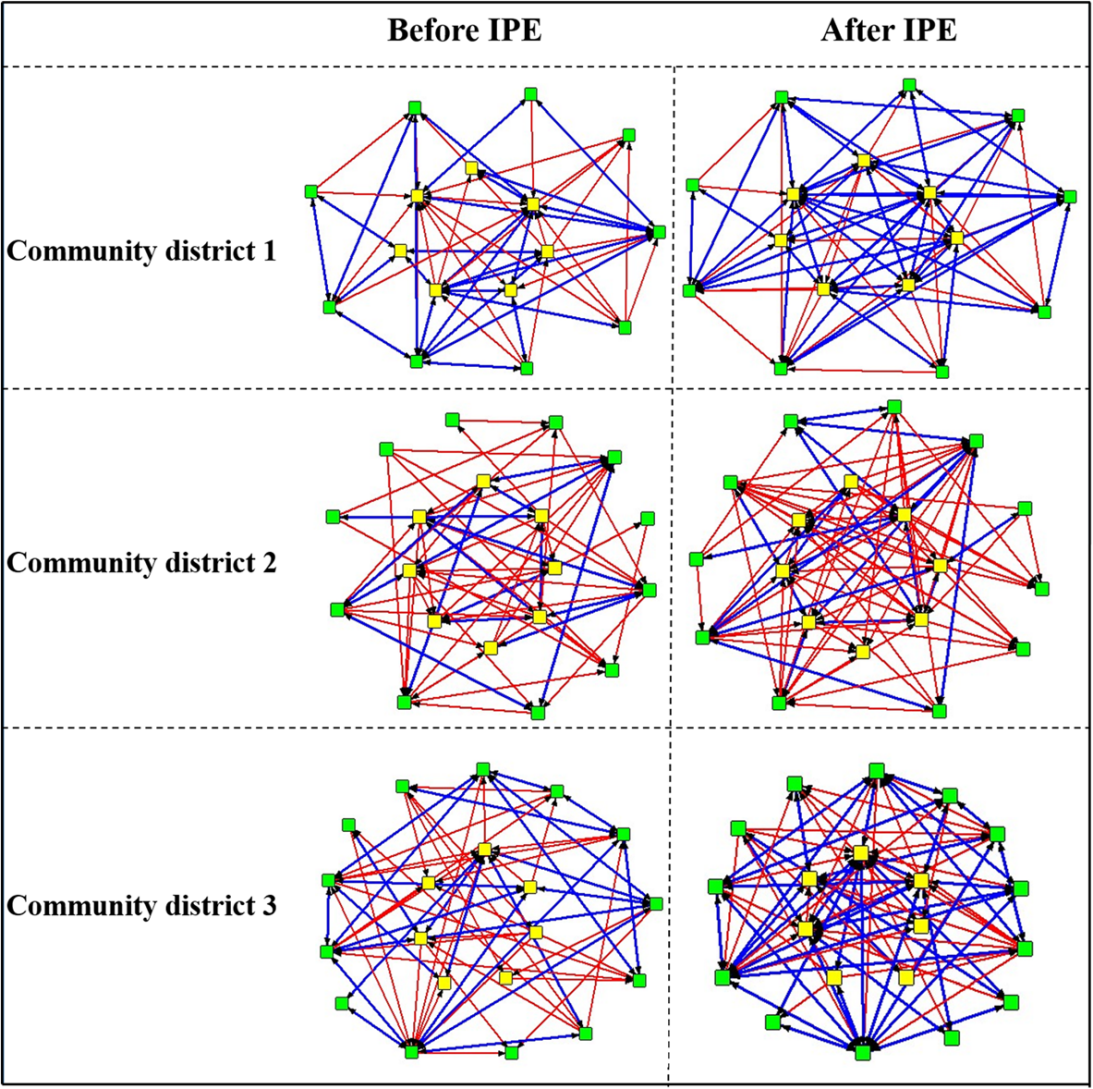
Community districts’ collaboration networks of reciprocal contacts. Blue lines, reciprocal contact; red lines, one-sided contact; yellow squares, healthcare professionals in ring 1; green squares, healthcare professionals in ring 2

Program participants and non-program participants had more reciprocal contacts after the IPCP program than before, which is shown in Fig. 4. The reciprocity increased over time with 15% in district 1, 2% in district 2, and 13% in district 3. In district 1, for example, before the IPCP program, 49% of all potential reciprocal relationships are actually reciprocal. This number increased to almost two-thirds (64%) after the IPCP program, indicating that the IPCP program also contributed to more sustained reciprocal collaborative efforts among the professionals.

### Diversity of contacts

The diversity of contacts increased over time, with almost 10% (CI −0.14 to −0.05, *p* value < 0.001) in district 1 and 6% in district 2 (CI −0.13 to −0.002, *p* value 0.055) and district 3 (CI −0.08 to 0.03, *p* value 0.371). For example, district 1 showed that of all possible diverse contacts, 32% of these were used before the IPCP program. The diversity of contacts, thus, the interprofessional collaboration between professionals, increased to 42% after the IPCP program. This finding suggests that the IPCP program contributed to a more diverse network of healthcare professionals, for both program participants as well as non-program participants.

### The value of contacts

Participants from district 2 valued their collaboration with other professionals significantly more after the IPCP program (*t* −2.35, CI −0.33 to −0.03, *p* value 0.022). However, this significant increase could not be confirmed for districts 1 (*t* −1.28, CI −0.28 to 0.06, *p* value 0.209) and 3 (*t* −1.12, CI −0.19 to 0.05, *p* value 0.267).

## Discussion

This study examined the feasibility of implementation of the IPCP program in three districts and evaluated its potential to increase interprofessional collaboration in caring for older people. First, our results indicate a high acceptability of the IPCP program as determined by the program participants. Second, the data collection as described in the “Methods” section showed potential to reach the healthcare professionals who were not participating in the program. Third, the social network analysis showed that it was possible to measure network development for each community district in which a spill-over effect was revealed. Compared with before the IPCP program, after the program, participants had a larger, more reciprocal, and more diverse interprofessional networks.

Our study showed that the IPCP program was found to be acceptable by the program participants. A more in depth understanding could be obtained when using validated questionnaires measuring levels 1, 2, and 3 of the adapted framework of Kirkpatrick for interprofessional education [22]. However, during the development process of the IPCP program, an expert team was involved to discuss the final content of the program, as previously described elsewhere [14]. The expert team did not have the scientific background to discuss the methodological rigor of the proposed evaluation but rather whether this evaluation seemed feasible for the program participants. The expert team stated that the content of the IPCP should not be at the expense of research purposes. The development team was therefore discouraged from including validated (multiple) questionnaires and therefore combined elements from the framework of Kirkpatrick for interprofessional education to one single questionnaire to examine the acceptability [22].

This study showed that it was feasible to collect social network data despite the lack of a clear network boundary. Because of scattered healthcare organizations in the districts, it was very difficult to generate a name roster of all professionals per district. These so-called “hidden,” fluid networks are difficult to reach [23], and it may mean that we did not include all potential professionals in the districts. However, by combining the snowball method and a fixed-list selection of names [19, 24], we sufficiently captured this “hidden” network to assess what we were interested in, namely, the feasibility of implementing an IPCP program to improve community collaboration networks.

This study showed that the interprofessional network of the participants of the IPCP program increased in size after the program. A larger network increases the likelihood of encountering actionable knowledge [25]. Moreover, not only did the program participants develop their interprofessional network but, the non-program participants did as well. This spill-over phenomenon reflects findings in other fields, e.g., educational science [26, 27], and can be explained by the theory of “three degrees of influence” [28]. Social influence, or the effect that the words and actions of others have on our thoughts, feelings, attitudes, or behavior, has the largest effect between people who are directly connected in a network (called “1 degree of separation”) [28]. Nonetheless, the theory of “three degrees of influence” asserts that social influence tends to ripple through our network to measurably influence others by up to three degrees of separation (colleague from a colleague of a colleague). While this may promote the adage “the bigger the network, the better,” this saying should be interpreted carefully. The likelihood of encountering actionable knowledge by increasing one’s network may be counteracted by the cost of maintaining a large network. In addition, the phrase ignores variety in the content and diversity of the network. Depending on what professionals need in caring for community-dwelling older people, some may benefit from increased network diversity while others may prefer a larger network. As such, in future work, network characteristics need to be studied in interaction. In addition, further research is necessary to understand how professionals with a large network versus those with a small network perceived the availability of actionable knowledge in their care for community-living older people.

The reciprocity in the district collaboration networks increased after the IPCP program. In other words, health and social care professionals tended to engage in more reciprocal connections after the IPCP program than before. Districts 1 and 3 showed a high increase in the reciprocity of contacts, and district 2 showed a low increase. District 2 also differed from the other districts with a lower reciprocity before the IPCP program. This study did not examine the underlying aspects for these changes. However, collaboration is a process that takes time and energy [11, 29]. Furthermore, it is a process in which several factors play a role, such as personal skills and attitudinal aspects, but just as important is the context in which professionals work together, that it is clear and balanced [29]. In addition, after the IPCP program, healthcare professionals in all three districts showed an increase in network diversity, as their networks consisted of multi-disciplinary professionals from the IPCP as well as outside the IPCP program. Network diversity is linked to opportunities for improvement as one can tap into different sources of expertise and experiences when framing complex care needs for community-living older people [30]. Although there was an increase in diversity, after the IPCP, the three community districts still only utilized 32-42% of the potential diversity in their network. One explanation could be that collaboration between professionals is still based on disorders that require specialist care instead of more integral and wellness-oriented care [31, 32].

In this study, the terms “collaboration” and “value” were not explicitly defined. The researchers have consciously made this choice because how professionals perceive and define collaboration can differ. For example, some professionals can value another professional because of their accessibility while for others, the fruitfulness of their contacts, regarding care for older people, is more important. This is in line with a concept analysis of interprofessional collaboration that demonstrates that IPC is a complex concept, which continues to evolve [5]. IPC has been studied as an outcome of IPE and as an antecedent to patient and provider outcomes. WHO stated that IPE has been proven to be essential in improving dynamics in local healthcare services [33]. Furthermore, coordinated home-based care by interprofessional teams is associated with lower consumption of care [34]. However, a Cochrane review from 2017 stated that there is not sufficient evidence to draw clear conclusions on the effects of IPC interventions for interprofessional practice and health outcomes, because of the certainty of evidence from the included studies, which was judged as low to very low [35]. Despite these inconclusive results regarding interprofessional practice and health outcomes, healthcare professionals are still in need of interprofessional educational programs to guide them to overcome the difficulties encountered by health professionals when collaborating in clinical practice to provide care to older people with complex care needs [35]. The outcomes of this feasibility study provided insights to expand this program on a larger scale. Following the Medical Research Council Framework, as this program can be seen as a complex intervention that contains several interacting components [36], the next step is to evaluate the IPCP program in regard to its (cost) effectiveness. A clear conceptualization of IPC, regarding antecedents, attributes, and outcomes of IPC in the context of primary care, is therefore first necessary for understanding interprofessional collaboration within different networks and how it may be strengthened [5].

### Strengths and limitations

This study is among the first that uses SNA to enrich common research methods to examine the feasibility of implementing an IPCP program. While SNA is an underused method within healthcare education and intervention design, it is a useful technique for examining how social relationships among professionals are established and evolved [37–41]. Another strength of this study is its use of different data sources (i.e., IPCP program participants as well as non-program participants) to examine the feasibility of implementing the IPCP program. This triangulation results in a low same-source and same-measurement-context (SS/SMC) bias, thereby increasing the validity of our study results [24, 39].

This study also has some limitations. First, the presence of a Hawthorne effect, the effect of an intervention that is solely due to intervention participation, cannot completely be excluded [42]. However, the non-program participants received no intervention, and a strong increase in contacts was also observed within this group indicating that the risk of the Hawthorne effect is limited. Moreover, the nature of this study was to examine the feasibility of implementation which is commonly done in uncontrolled settings [42]. Second, the small number of participants limits generalizability. However, a large body of research has found that SNA techniques provide a robust insight into actual social networks, as these techniques focus on relationships rather than individuals [43–47]. Third, this study observed unit non-response defined as missing healthcare professionals in which all outgoing contacts of a professional are missing but not the incoming contacts. Although non-response results in missed contacts for some actors, partial information on the network context of the incompletely observed professionals was available due to their responding colleagues [19]. This information was included in this study and expressed within the “Results” section as ring 3 to provide a comprehensive understanding of the collaboration networks.

## Conclusion

This study showed that it was feasible to implement an IPCP program in terms of acceptability, feasibility of data collection and social network analysis, and to measure network development in order to see the potential of the IPCP program to increase interprofessional collaboration between primary healthcare professionals in caring for the older population. After the IPCP program, the program participants as well as non-program participants gained a larger more collaborative, and diverse interprofessional network in primary care, suggesting a spill-over effect of networked interventions. Future studies are needed to determine the effects of interprofessional collaboration on continuity of care as well as its cost-savings.

## Supplementary Information

**Additional file 1.** Evaluation questions IPCP program.

## Data Availability

The datasets used and/or analyzed during the current study are available from the corresponding author on reasonable request.
