# Supplementary material for: Implementation of an interprofessional collaboration in practice program: a feasibility study using social network analysis
Source: Pilot Feasibility Stud. 2021 Jan 6;7:7. doi: 10.1186/s40814-020-00746-3 (PMC7786471; doi:10.1186/s40814-020-00746-3)
Supplement: Supplementary file 1 — Additional file 1. Evaluation questions IPCP program. [file 40814_2020_746_MOESM1_ESM.docx]

**Additional file 1**. **Evaluation questions IPCP program**

|  | | **Total**  (N=22)  **N (%)** | **District 1**  (N=7)  **N (%)** | **District 2**  (N=8)  **N (%)** | **District 3**  (N=7)  **N (%)** |
| --- | --- | --- | --- | --- | --- |
| 1 | Did you find the content of this IPCP program instructive  *□ Yes*  *□ A little*  *□ No* | 18 (81.8)  4 (18.2)  0 (0.0) | 5 (71.4)  2 (28.6)  0 (0.0) | 7 (87.5)  1 (12.5)  0 (0.0) | 6 (85.7)  1 (14.3)  0 (0.0) |
| 2 | How do you score the content of the offered knowledge / skills in the IPCP program?  *Mean (sd)* | 7.69 (0.65) | 7.79 (0.65) | 7.69 (0.66) | 7.58 (0.61) |
| 3 | Did the IPCP program contribute to enhanced interprofessional collaboration?  *Mean (sd)* | 7.69 (0.63) | 7.86 (0.64) | 7.67 (0.66) | 7.50 (0.46) |
| 4 | Did you find the connection between the components of this IPCP program good.  *□ Yes*  *□ A little*  *□ No* | 20 (90.9)  2 (9.1)  0 (0.0) | 7 (100.0)  0 (0.0)  0 (0.0) | 8 (100.0)  0 (0.0)  0 (0.0) | 5 (71.4)  2 (28.6)  0 (0.0) |
| 5 | Did you find the assignments to be carried out instructive in the context of this IPCP program?  *□ Yes*  *□ A little*  *□ No* | 15 (68.2)  7 (31.8)  0 | 6 (85.7)  1 (14.3)  0 (0.0) | 6 (75.0)  2 (25.0)  0 (0.0) | 3 (42.9)  4 (57.1)  0 (0.0) |
| 6 | Were the learning objectives of this IPCP program clear?  *□ Yes*  *□ A little*  *□ No* | 15 (68.2)  7 (31.8)  0 (0.0) | 5 (71.4)  2 (28.6)  0 (0.0) | 5 (62.5)  3 (37.5)  0 (0.0) | 5 (71.4)  2 (28.6)  0 (0.0) |
| 7 | Did you start acting differently towards your fellow professionals after this IPCP program, in terms of role identity?  *□ Yes*  *□ A little*  *□ No* | 8 (36.4)  8 (36.4)  6 (27.3) | 4 (57.1)  2 (28.6)  1 (14.3) | 3 (37.5)  3 (37.5)  2 (25.0) | 3 (42.9)  3 (42.9)  1 (14.2) |
| 8 | Are you sufficiently able to apply in practice what you have learned from the IPCP program.  *□ Yes*  *□ A little*  *□ No* | 15 (68.2)  6 (27.3)  1 (4.6) | 5 (71.4)  1 (14.3)  1 (14.3) | 6 (75.0)  2 (25.0)  0 (0.0) | 4 (57.1)  3 (42.9)  0 (0.0) |
| 9 | What did you think of the organization of this IPCP program?  *□ Insufficient □ Sufficient □ More than sufficient □ Good □ Very good* | 0 (0.0)  4 (18.2)  4 (18.2)  13 (59.1)  1 (4.6) | 0 (0.0)  0 (0.0)  1 (14.3)  6 (85.7)  0 (0.0) | 0 (0.0)  0 (0.0)  3 (37.5)  4 (50.0)  1 (12.5) | 0 (0.0)  4 (57.1)  0 (0.0)  3 (42.9)  0 (0.0) |
| 10 | What did you think of the accessibility of the IPCP program coordinator?  *□ Insufficient □ Sufficient □ More than sufficient □ Good □ Very good* | 0 (0.0)  2 (9.1)  1 (4.6)  16 (72.7)  2 (9.1) | 0 (0.0)  0 (0.0)  0 (0.0)  6 (85.7)  1 (14.3) | 0 (0.0)  0 (0.0)  1 (12.5)  6 (75.0)  1 (12.5) | 0 (0.0)  2 (28.6)  1 (14.3)  4 (57.1)  0 (0.0) |
| 11 | Does the teaching material on the online platform provide complete information?  *□ Yes*  *□ A little*  *□ No* | 7 (31.8)  12 (54.6)  3 (13.6) | 3 (42.9)  4 (57.1)  0 (0.0) | 4 (50.0)  4 (50.0)  0 (0.0) | 0 (0.0)  4 (57.1)  3 (42.9) |
| 12 | Is the teaching material clearly written (linguistic)?  *□ Yes*  *□ A little*  *□ No* | 17 (77.3)  4 (18.2)  1 (4.6) | 5 (71.4)  1 (14.3)  1 (14.3) | 8 (100.0)  0 (0.0)  0 (0.0) | 4 (57.1)  3 (42.9)  0 (0.0) |
| 13 | Are you satisfied with the order in which the teaching material is covered in the IPCP program?  *□ Yes*  *□ A little*  *□ No* | 20 (90.9)  2 (9.1)  0 (0.0) | 7 (100.0)  0 (0.0)  0 (0.0) | 8 (100.0)  0 (0.0)  0 (0.0) | 5 (71.4)  2 (28.6)  0 (0.0) |
| 14 | Connect the content and form of the lesson to your learning needs  (Think of steering, guidance, independence).  *□ Yes*  *□ A little*  *□ No* | 15 (68.2)  6 (27.3)  0 (0.0) | 5 (71.4)  2 (28.6)  0 (0.0) | 7 (87.5)  1 (12.5)  0 (0.0) | 3 (42.9)  3 (42.9)  1(14.3) |
| 15 | Was the trainer well informed about the setup of this IPCP program?  □ *Insufficient □ Sufficient □ More than sufficient □ Good □ Very good* | 0 (0.0)  2 (9.1)  2 (9.1)  13 (59.1)  5 (22.7) | 0 (0.0)  0 (0.0)  0 (0.0)  5 (71.4)  2 (28.6) | 0 (0.0)  0 (0.0)  0 (0.0)  6 (75.0)  2 (25.0) | 0 (0.0)  2 (28.6)  2 (28.6)  2 (28.6)  1 (14.3) |
| 16 | Was the trainer enthusiastic?  *□ Insufficient □ Sufficient □ More than sufficient □ Good □ Very good* | 0 (0.0)  0 (0.0)  2 (9.1)  4 (63.6)  6 (27.3) | 0 (0.0)  0 (0.0)  0 (0.0)  6 (85.7)  1 (14.3) | 0 (0.0)  0 (0.0)  0 (0.0)  4 (50.0)  4 (50.0) | 0 (0.0)  0 (0.0)  2 (28.6)  4 (57.1)  1 (14.3) |
| 17 | Did the trainer motivate the trainees? □ *Insufficient □ Sufficient □ More than sufficient □ Good □ Very good* | 0 (0.0)  1 (4.6)  2 (9.1)  13 (59.1)  6 (27.3) | 0 (0.0)  0 (0.0)  0 (0.0)  5 (71.4)  2 (28.6) | 0 (0.0)  0 (0.0)  0 (0.0)  5 (62.5)  3 (37.5) | 0 (0.0)  1 (14.3)  2 (28.6)  3 (42.9)  1 (14.3) |
| 18 | Did the trainer provide sufficient information when needed?  □ *Yes*  *□ A little*  *□ No* | 22 (100.0)  0 (0.0)  0 (0.0) | 7 (100.0)  0 (0.0)  0 (0.0) | 8 (100.0)  0 (0.0)  0 (0.0) | 7 (100.0)  0 (0.0)  0 (0.0) |
| 19 | How do you score the trainer? Score 0-10  *Mean (sd)* | 8.18 (0.57) | 8.14 (0.64) | 8.38 (0.48) | 8.00 (0.53) |
| 20 | How do you rate the IPCP program in general? Score 0-10  *Mean (sd)* | 7.64 (0.62) | 7.43 (0.49) | 7.94 (0.68) | 7.50 (0.46) |
